# Association of lipoprotein levels with mortality in subjects aged 50 + without previous diabetes or cardiovascular disease: A population-based register study

**DOI:** 10.3109/02813432.2013.824157

**Published:** 2013-09

**Authors:** Lise Bathum, René Depont Christensen, Lars Engers Pedersen, Palle Lyngsie Pedersen, John Larsen, Jørgen Nexøe

**Affiliations:** ^1^Department of Clinical Biochemistry, Slagelse Hospital, Region Zealand, Denmark; ^2^Institute of Regional Health Services Research, University of Southern Denmark, Denmark; ^3^Research Unit of General Practice, Institute of Public Health, University of Southern Denmark, Odense, Denmark

**Keywords:** Cholesterol, Denmark, epidemiology, general practice, lipids, lipoproteins, mortality

## Abstract

**Objective:**

This study aimed to investigate the association of lipoprotein and triglyceride levels with all-cause mortality in a population free from diabetes and cardiovascular disease (CVD) at baseline. The European Guidelines on cardiovascular disease prevention state that in general total cholesterol (TC) should be < 5 mmol/L (190 mg/dL) and low-density lipoprotein cholesterol (LDL-C) should be < 3 mmol/L (115 mg/dL).

**Design:**

A population-based register study in the period 1999–2007 including 118 160 subjects aged 50 + without statin use at baseline. All-cause mortality was related to lipoprotein and triglyceride levels and adjusted for statin use after inclusion.

**Results:**

All-cause mortality was lower in the groups with TC or LDL-C above the recommended levels. Compared with subjects with TC < 5 mmol/L, adjusted hazard ratios for the group aged 60–70 years ranged from 0.68 (95% confidence interval (CI) 0.61–0.77) for TC 5–5.99 mmol/L to 0.67 (95% CI 0.59–0.75) for TC 6–7.99 mmol/L and 1.02 (95% CI 0.68–1.53) for TC ≥ 8 mmol/L in males and from 0.57 (95% CI 0.48–0.67) to 0.59 (95% CI 0.50–0.68) and 1.02 (95% CI: 0.77–1.37) in females. For triglycerides, ratios compared with the group < 1 mmol/L in the females aged 60–70 years ranged from 1.04 (95% CI 0.88–1.23) to 1.35 (95% CI 1.10–1.66) and 1.25 (95% CI 1.05–1.48) for triglycerides 1–1.39 mmol/L, 1.4–1.69 mmol/L, and ≥ 1.7 mmol/L, respectively. Statin treatment after inclusion provided a survival benefit.

**Conclusion:**

These associations indicate that high lipoprotein levels do not seem to be definitely harmful in the general population. However, high triglyceride levels in females are associated with decreased survival.

There is an intense debate as to whether the recommended target levels from the lipid- lowering guidelines provide a survival difference in primary prevention. More than 50% of the population aged 50 + have levels above the recommended levels.

- This study indicates that high total, HDL-, or LDL-cholesterol in those aged 50 + years free from CVD or diabetes at baseline are associated with lower all-cause mortality.
- However, cholesterol-lowering treatment in the form of statins provides a survival benefit without correlation to cholesterol level.
- High triglyceride levels were found to be associated with higher all-cause mortality, which was most pronounced in women.

## Introduction

Total cholesterol (TC) is a well-documented risk factor for cardiovascular and all-cause mortality [1–3]. The causal relationship between TC and cardiovascular disease (CVD) is furthermore well recognized [4]. The evidence that reducing TC reduces the risk of cardiovascular disease is considered equally unequivocal [3,4]. The European Guidelines on Cardiovascular Disease Prevention in Clinical Practice [4] state that in general plasma TC should be below 5 mmol/L (190 mg/dL) and low-density lipoprotein cholesterol (LDL-C) should be below 3 mmol/L (115 mg/dL). In the highest risk subjects, especially those with clinically established CVD or diabetes, the treatment goals should be lower. The European guidelines do not define a specific target level for HDL cholesterol (HDL-C) or triglycerides, but concentrations of HDL-C < 1 mmol/L (˜40 mg/dL) in men and 1.2 mmol/L (45 mg/dL) in women, and, similarly, fasting triglycerides > 1.7 mmol/L (150 mg/dL) serve as markers of CVD risk[4]. Similar guidelines, although not as specific in low-risk subjects, are issued by the Canadian Cardiovascular Society and the American National Cholesterol Education Program [5].

The Danish guidelines on CVD prevention prepared by the Danish Society of Cardiology as part of the Danish national cardiological treatment guidelines (http://nbv.cardio.dk/) and updated in May 2012, states that primary prevention should be considered in individuals with multiple risk factors (≥ 5% within 10 years), type 2 diabetes, or type 1 diabetes with high risk (nephropathy, proliferative eye disease, or 40 years with several risk factors), considerable increased single-risk factor or close relative with premature CVD or very high CVD risk. To estimate the risk, the European cardiovascular disease risk assessment model, the SCORE Risk Charts, is used developed by the European Society of Cardiology [6]. Denmark, as well as the other Scandinavian countries, is now considered at low risk for CVD and use of the low-risk chart is therefore recommended. These charts estimate a risk based on gender, age, total cholesterol, systolic blood pressure, and smoking status. With an estimated risk above 5% medical treatment is recommended and at a risk above 10% treatment is strongly recommended.

The Nordic Reference Interval Project (NORIP), a project with the goal of recommending Nordic plasma reference intervals for the most common quantities used in clinical chemistry, shows that the values for TC and LDL-C increase with age and that the central 95% reference intervals for subjects aged 50 + are for TC 3.9–7.8 mmol/L (151–302 mg/dL) and for LDL-C 2.5–5.3 mmol/L (97–205 mg/dL) [7]. HDL-C and triglycerides are not markedly age-related and have the reference intervals 1.0–2.7 mmol/L (39 – 104 mg/dL) and 0.45–2.6 mmol/L (40–230 mg/dL), respectively [7]. As TC, HDL-C, and LDL-C are approximately normally distributed, this means that more than 50% of all subjects aged 50 + in the Nordic countries have TC and LDL-C above the recommended levels.

There is some evidence that age attenuates the relative effect of TC on the risk of CVD and ischaemic heart disease mortality [2,8] and most studies that find a relationship between TC and all-cause mortality have been performed in the middle-aged. Several studies in the elderly have reported that TC has a strong inverse association with non-CVD mortality [9–14], meaning that people with higher levels of TC have a lower risk of non-CVD mortality. Low cholesterol levels could be due to the cholesterol-lowering effect of chronic diseases and inflammatory processes [15].

The benefit of use of statins (HMG-CoA reductase inhibitors) in patients with CVD or diabetes is well established and documented [16], but statin use for primary prevention of CVD is still controversial [17–22]. According to the lipid-lowering guidelines [4,5] and the established NORIP reference intervals [7], more than half of the population aged 50 + years does have TC and LDL-C above the target levels and should therefore be encouraged to adopt lifestyle modifications and perhaps statin therapy. A lipoprotein measurement above the recommended targets could provoke unwarranted fear in subjects with such a value. As an aid in clinical counselling, it seems important to clarify the relation between TC and its subfractions and mortality.

In order to investigate whether the recommended target levels from the lipid-lowering guidelines provide a survival difference in the general population at baseline, we conducted a population-based register study in the period 1999–2007 including 118 160 subjects aged 50 + with a TC measurement without a diagnosis of diabetes or CVD prior to the measurement. We tested all-cause mortality in this population and related it to lipoprotein and triglyceride levels, education, and use of statins.

## Material and methods

This study is based on lipoprotein and triglyceride measurements from the populations in two former Danish counties, the West Zealand and Storstrom Counties, now part of Region Zealand. These two counties had a population of 583 917 citizens on 1 January 2007. Data were drawn from the Laboratory Information Management System (LIMS) in these two counties that manages all data from biochemical measurements in the population of the two counties, independently of referring doctor. Recorded information includes the 10-digit Civil Personal Registration number (CPR) unique to every Danish resident. The CPR number was used to link information on all patients above 18 years between the different registries.

### Biochemical cholesterol measurements

Enzymatic methods (Roche, Basel, Switzerland) were routinely used on fresh samples to measure the levels of TC, HDL-C, and triglycerides. LDL-C was estimated from the Friedewald equation:


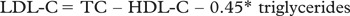


LDL-C was not estimated if triglycerides were above 4.4 mmol/L. Triglycerides and LDL-C were only obtained in the fasting state until 20 November 2007. After this date the triglyceride and LDL-C measurement is a mixture of fasting and non-fasting values as it was shown that lipid profiles change only minimally in response to food intake [23].

### Information regarding CVD, diabetes, cholesterol-lowering treatment, and education

The information regarding CVD was obtained from the Danish National Registry of Patients, which includes information on 99.4% of all discharges from non-psychiatric hospitals in Denmark. From 1977 to 1994 the registry included hospital discharges. Ambulatory visits and visits to casualty departments were included from 1995. Recorded information includes the CPR number, the dates on admission and discharge, and up to 20 discharge diagnoses, classified according to the Danish version of the International Classification of Diseases, Tenth revision (ICD10). For this study, we retrieved data regarding ischaemic heart disease and stroke (ICD10: I20–I25; I60–66) and arteriosclerosis (ICD10 I70–I74) from the period 1989–2007. CVD patients were in this study defined as subjects with one or more of these diagnosis codes in this period.

The information regarding diabetes was obtained from the Danish National Diabetes Register. It contains information regarding subjects with a diagnosis of diabetes in the period 1996 to 2008. Data regarding cholesterol-lowering treatment in the form of statins were obtained from the Register of Medicinal Product Statistics in the period 1998–2008. Statin use is defined as one prescription (ATC C10AA) any time in the period from inclusion to end of study period.

Data regarding education were obtained from Statistics Denmark.

All subjects with a TC measurement in the period from 13 September 1999 to 31 December 2007, surviving at least one year after the initial TC measurement, were included. Thus, end of study period was 31 December 2008. Subjects with a diagnosis of CVD or diabetes prior to or 14 days after the initial TC measurement were excluded. A total of 12 992 and 571 subjects had a diagnosis of diabetes or CVD, respectively, later than 14 days after the TC measurement. They remained in the study. As statins affect the cholesterol level, all subjects with at least one prescription of statin in the year prior to the including TC measurement were excluded from the study.

### Ethical considerations

The study was approved by the Danish Data Protection Agency (number: 2008-58-0020).

### Statistical analysis

For the survival analysis, participants were followed from the date of their first cholesterol measurement until emigration, death, or end of study period (31 December 2008). Information on emigration and death was retrieved from the Danish Civil Registration System (CRS), which is continuously updated. The first registered measurement was used and the date of blood sampling was the date of inclusion. TC, LDL-C, HDL-C, and triglycerides were analysed in groups, mainly based on the recommendations from the European Guidelines on Cardiovascular Disease Prevention in Clinical Practice [4].

TC was stratified into four categories (< 5 mmol/L (190 mg/dL), 5–5.99 mmol/L (190–232 mg/dL), 6–7.99 mmol/L (232–309 mg/dL) and ≥ 8 mmol/L (≥ 309 mg/dL)), HDL-C into four categories (< 1 mmol/L (< 39 mg/dL), 1–1.49 mmol/L (39–58 mg/dL), 1.5–1.99 mmol/L (58–77 mg/dL) and ≥ 2 mmol/L (≥ 77 mg/dL)), LDL cholesterol into four categories (< 2.5 mmol/L (< 97 mg/dL), 2.5–2.99 mmol/L (97–116 mg/dL), 3.0–3.99 mmol/L (116–155 mg/dL) and ≥ 4.0 mmol/L (≥ 155 mg/dL), and triglycerides into four categories (< 1 mmol/L (< 89 mg/dL), 1–1.39 mmol/L (89–132 mg/dL), 1.4–1.69 mmol/L (132–150 mg/dL) and ≥ 1.7 mmol/L (≥ 151 mg/dL). The conversion factor between mmol/L and mg/dL is for TC, HDL-C, and LDL-C 38.67 and for triglycerides 88.57.

Survival was estimated by the Kaplan–Meier method and analysed using the Cox proportional-hazards model. As the different lipoproteins are correlated, they were tested in separate analyses, and as the lipoprotein levels are dependent on age and sex, all analyses were performed separately for males and females as well as in age intervals. Furthermore, to ensure that low TC was not due to terminal cancer or hepatic disease, only subjects with a minimum follow-up period of one year after initial measurements were included, thus excluding subjects with terminal disease. We tested for the potential interaction between statin use and TC, LDL-C, HDL-C, triglyceride, and educational levels, respectively, using the Wald test. In the case of significant findings, the relevant analyses were adjusted accordingly.

## Results

A total of 173 484 subjects aged 50 + years at measurement had TC, HDL-C, LDL-C, and triglyceride measurement performed in the period 1999–2007. Before data analysis, all subjects with a diagnosis of diabetes (16 150 subjects) or CVD (2238 subjects) previous to or 14 days after the measurement and subjects with a prescription of statin during the last year before test date (10 917 subjects) were excluded. Several individuals had more than one reason for exclusion, so the total was 25 621 subjects. Furthermore, we excluded subjects who died within the first year after inclusion (5281 subjects) and subjects with less than one year of follow-up (16 461). The study population then comprised 126 121 subjects aged 50–103 years. Of these, 38 525 subjects (30.6%) had a minimum of one statin prescription after inclusion.

School education was divided into “less than 12 years” (42.5%), “between 12 and 15 years” (48.5%), and “more than 15 years” (2.7%). Information was lacking in 7961 subjects, who were therefore excluded, resulting in a total of 118 160 subjects for statistical analysis.

The proportional hazard assumption was met for the survival analyses. The median survival time was 8.6, 8.5, and 7.3 years for the age groups 50–60, 60–70, and > 70 years, respectively.

Results from the survival analyses are given in Tables I–IV. For TC, the mortality was significantly lower in the TC groups 5–5.99 mmol/L and 6–7.99 mmol/L, in both sexes and in all age groups compared with the group with TC < 5 mmol/L. For the groups with the highest TC level (≥ 8 mmol/L), the mortality becomes significantly lower in the oldest age group compared with the group with the lowest TC. For HDL-C in females, there was a decreased mortality in all ages in the groups with a HDL-C above 1 mmol/L. In males there was a decreased mortality in the HDL-C groups 1–1.49 and 1.5–1.99 mmol/L in all age groups, but the highest HDL-C group only had a decreased mortality in the age group > 70 years.

**Table I.** Mortality risk according to TC derived from the Cox regression analysis shown as the hazard ratio (HR), p-value and the 95% confidence interval (95% CI).

		HR	p-value	95% CI		HR	p-value	95% CI
Parameter	n	Males			n	Females		
50–60 years		N = 25 840; C = 1572				N = 25 743; C = 866		
TC < 5 mmol/L^1^	5450				3957			
TC 5–5.99 mmol/L	9100	0.73	< 0.001	0.64–0.82	8,637	0.68	< 0.001	0.56–0.82
TC 6–7.99 mmol/L	10 278	0.68	< 0.001	0.59–0.77	11 682	0.71	< 0.001	0.59–0.86
TC ≥ 8 mmol/L	1012	1.07	0.601	0.83–1.37	1467	1.00	0.977	0.74–1.36
Statin	7373	0.62	< 0.001	0.55–0.70	6835	0.63	< 0.001	0.53– 0.74
School 12–15 years		0.63	< 0.001	0.57–0.69		0.66	< 0.001	0.58–0.76
School ≥ 15 years		0.44	< 0.001	0.30–0.63		0.60	0.038	0.37–0.97
60–70 years^2^		N = 19 843; C = 2379				N = 20 367; C = 1641		
TC < 5 mmol/L^1^	4047				2170			
TC 5–5.99 mmol/L	7161	0.68	< 0.001	0.61–0.77	5795	0.57	< 0.001	0.48–0.67
TC 6–7.99 mmol/L	8001	0.67	< 0.001	0.59–0.75	10 809	0.59	< 0.001	0.50–0.68
TC ≥ 8 mmol/L	634	1.02	0.914	0.68–1.53	1593	1.02	0.866	0.77–1.37
Statin	6971	0.67	< 0.001	0.55–0.83	7214	0.66	0.029	0.46–0.96
School 12–15 years		0.81	< 0.001	0.74–0.88		0.72	< 0.001	0.65–0.80
School ≥ 15 years		0.53	< 0.001	0.40–0.71		0.81	0.204	0.59–1.12
> 70 years		N = 11 685; C = 3503				N = 14 682; C = 3039		
TC < 5mmol/L^1^	3,183				1,789			
TC 5–5.99 mmol/L	4,308	0.70	< 0.001	0.65–0.76	4,207	0.65	< 0.001	0.58–0.72
TC 6–7.99 mmol/L	3,956	0.62	< 0.001	0.57–0.68	7,581	0.52	< 0.001	0.47–0.57
TC ≥ 8 mmol/L	238	0.67	0.001	0.52–0.86	1,105	0.59	< 0.001	0.50–0.69
Statin	3,675	0.64	< 0.001	0.59–0.69	4,613	0.60	< 0.001	0.55–0.65
School 12–15 years		0.84	< 0.001	0.78–0.90		0.84	< 0.001	0.78–0.92
School ≥ 15 years		0.79	0.022	0.65–0.97		0.82	0.179	0.61–1.10

Notes: N = numbers in the specific age and sex group, C = cases (died in the study period more than one year after inclusion), and n = numbers in each TC group or numbers receiving statin after inclusion. ^1^The TC group < 5 mmol/L is the reference group and only the numbers in this group are therefore reported. ^2^The results shown are adjusted for the found interaction between TC and statin use in both males and females aged 60–70 years.

**Table II.** Mortality risk according to HDL-C derived from the Cox regression analysis shown as the hazard ratio (HR), p-value and the 95% confidence interval (95% CI).

		HR	p-value	95% CI		HR	p-value	95% CI
Parameter	n	Males			n	Females		
50–60 years		N = 23 711; C = 1361				N = 23 818; C = 754		
HDL-C < 1 mmol/L^1^	3270				906			
HDL-C 1–1.49 mmol/L	12 743	0.65	< 0.001	0.56–0.75	7726	0.53	< 0.001	0.40–0.69
HDL-C 1.5–1.99 mmol/L	5935	0.64	< 0.001	0.54–0.76	9677	0.40	< 0.001	0.30–0.53
HDL-C ≥ 2 mmol/L	1763	1.15	0.168	0.94–1.40	5509	0.47	< 0.001	0.35–0.63
Statin	6702	0.58	< 0.001	0.51–0.66	6356	0.62	< 0.001	0.53–0.74
School 12–15 years		0.62	< 0.001	0.56–0.69		0.72	< 0.001	0.63–0.84
School ≥ 15 years		0.42	< 0.001	0.28–0.62		0.64	0.092	0.38–1.07
60–70 years^2^		N = 18 230; C = 2105				N = 18 954; C = 1428		
HDL-C < 1 mmol/L^1^	1,984				626			
HDL-C 1–1.49 mmol/L	9270	0.67	< 0.001	0.59–0.76	5899	0.41	< 0.001	0.32–0.52
HDL-C 1.5–1.99 mmol/L	5347	0.57	< 0.001	0.50–0.66	7616	0.35	< 0.001	0.27–0.45
HDL-C ≥ 2 mmol/L	1629	0.91	0.266	0.77–1.08	4813	0.40	< 0.001	0.31–0.52
Statin	6327	0.55	< 0.001	0.50–0.60	6737	0.28	< 0.001	0.17–0.44
School 12–15 years		0.82	< 0.001	0.75–0.89		0.73	< 0.001	0.65–0.81
School ≥ 15 years		0.52	< 0.001	0.38–0.71		0.88	0.421	0.63–1.21
> 70 years		N = 10 302; C = 2952				N = 12 937; C = 2543		
HDL-C < 1 mmol/L^1^	1147				459			
HDL-C 1–1.49 mmol/L	5163	0.69	< 0.001	0.62–0.77	3940	0.60	< 0.001	0.51–0.72
HDL-C 1.5–1.99 mmol/L	3032	0.65	< 0.001	0.58–0.73	5134	0.54	< 0.001	0.45–0.64
HDL-C ≥ 2 mmol/L	960	0.80	0.004	0.69–0.93	3404	0.50	< 0.001	0.42–0.59
Statin	3248	0.58	< 0.001	0.54–0.63	4120	0.53	< 0.001	0.48–0.58
School 12–15 years		0.85	< 0.001	0.79–0.92		0.86	0.002	0.79–0.95
School ≥ 15 years		0.80	0.048	0.65–1.00		0.76	0.106	0.55–1.06

Notes: N = total numbers in the specific age and sex group, C = cases (died in the study period more than one year after inclusion) and n = numbers in each HDL-C or numbers receiving statin after inclusion. ^1^HDL-C group < 1 mmol/L is the reference group and only the numbers in this group are therefore reported. ^2^The results shown are adjusted for the found interaction between statin use and HDL-C in females aged 60–70 years.

**Table III.** Mortality risk according to LDL-C derived from the Cox regression analysis shown as the hazard ratio (HR), p-value and the 95% confidence interval (95% CI).

		HR	p-value	95% CI		HR	p-value	95% CI
Parameter	n	Males			n	Females		
50–60 years		N = 17,447; C = 950				N = 17,818; C = 519		
LDL-C < 2.5 mmol/L^1^	1601				1558			
LDL-C 2.5–2.99 mmol/L	2191	0.57	< 0.001	0.46–0.72	2300	0.62	0.008	0.44–0.88
LDL-C 3–3.99 mmol/L	6871	0.44	< 0.001	0.37–0.54	6973	0.58	< 0.001	0.44–0.77
LDL-C ≥ 4 mmol/L	6784	0.44	< 0.001	0.37–0.54	6987	0.69	0.010	0.51–0.91
Statin	4995	0.66	< 0.001	0.57–0.77	4798	0.69	< 0.001	0.56–0.85
School 12–15 years		0.63	< 0.001	0.55–0.72		0.67	< 0.001	0.57–0.80
School ≥ 15 years		0.57	0.008	0.38–0.86		0.58	0.093	0.31–1.09
60–70 years		N = 13 733; C = 1507				N = 14 298; C = 1023		
LDL-C < 2.5 mmol/L^1^	1183				949			
LDL-C 2.5–2.99 mmol/L	1647	0.67	< 0.001	0.56–0.81	1453	0.56	< 0.001	0.43–0.72
LDL-C 3–3.99 mmol/L	5492	0.49	< 0.001	0.42–0.57	5182	0.45	< 0.001	0.37–0.55
LDL-C ≥ 4 mmol/L	5411	0.45	< 0.001	0.38–0.53	6714	0.47	< 0.001	0.38–0.57
Statin	4872	0.63	< 0.001	0.56–0.71	5216	0.60	< 0.001	0.52–0.69
School 12–15 years		0.82	< 0.001	0.74–0.91		0.67	< 0.001	0.59–0.76
School ≥ 15 years		0.48	< 0.001	0.33–0.71		0.64	0.047	0.42–0.99
> 70 years		N = 7493; C = 2090				N = 9142; C = 1711		
LDL-C < 2.5 mmol/L^1^	874				717			
LDL-C 2.5–2.99 mmol/L	1067	0.71	< 0.001	0.61–0.83	985	0.66	< 0.001	0.54–0.80
LDL-C 3–3.99 mmol/L	2995	0.60	< 0.001	0.53–0.68	3310	0.52	< 0.001	0.44–0.60
LDL-C ≥ 4 mmol/L	2557	0.52	< 0.001	0.46–0.60	4130	0.46	< 0.001	0.40–0.54
Statin	2543	0.63	< 0.001	0.57–0.70	3098	0.62	< 0.001	0.55–0.69
School 12–15 years		0.81	< 0.001	0.75–0.89		0.82	< 0.001	0.73–0.91
School ≥ 15 years		0.69	0.005	0.53 –0.89		0.66	0.055	0.43–1.01

Notes: N = total numbers in the specific age and sex group, C = cases (died in the study period more than one year after inclusion) and n = numbers in each LDL-C or numbers receiving statin after inclusion. ^1^LDL-C group < 2.5 mmol/L is the reference group and only the numbers in this group are therefore reported.

**Table IV.** Mortality risk according to triglycerides derived from the Cox regression analysis shown as the hazard ratio (HR), p-value and the 95% confidence interval (95% CI).

		HR	p-value	95% CI		HR	p-value	95% CI
Parameter	n	Males			n	Females		
50–60 years		N = 18 207; C = 1001				N = 18 210; C = 544		
Trig < 1 mmol/L^1^	4390				5936			
Trig 1–1.39 mmol/L	4968	0.92	0.337	0.77–1.10	5561	1.34	0.017	1.05–1.71
Trig 1.4 –1.69 mmol/L	1907	0.83	0.125	0.65–1.05	1836	1.56	0.005	1.15–2.13
Trig ≥ 1.7 mmol/L	6942	1.02	0.809	0.87–1.20	4877	1.96	< 0.001	1.55 –2.48
Statin	5402	0.59	< 0.001	0.51–0.68	5002	0.58	< 0.001	0.48–0.71
School 12–15 years		0.62	< 0.001	0.54–0.70		0.69	< 0.001	0.58–0.82
School ≥ 15 years		0.54	0.003	0.36–0.81		0.60	0.108	0.32–1.12
60–70 years		N = 14 079; C = 1560				N = 14 537; C = 1057		
Trig < 1 mmol/L^1^	3826				4004			
Trig 1–1.39 mmol/L	4328	1.10	0.167	0.96–1.26	4661	1.04	0.651	0.88–1.23
Trig 1.4 –1.69 mmol/L	1591	1.01	0.935	0.84–1.21	1607	1.35	0.005	1.10–1.66
Trig ≥ 1.7 mmol/L	4334	1.15	0.039	1.01–1.32	4265	1.25	0.010	1.05–1.48
Statin	5066	0.56	< 0.001	0.50–0.62	5354	0.52	< 0.001	0.46–0.60
School 12–15 years		0.81	< 0.001	0.73–0.89		0.69	< 0.001	0.61–0.79
School ≥ 15 years		0.50	< 0.001	0.34–0.72		0.69	0.092	0.45–1.06
> 70 years		N = 7612; C = 2134				N = 9266; C = 1738		
Trig < 1 mmol/L^1^	2480				2256			
Trig 1–1.39 mmol/L	2518	0.97	0.582	0.87–1.08	3223	1.08	0.235	0.95–1.23
Trig 1.4–1.69 mmol/L	788	0.91	0.215	0.78–1.06	1162	1.17	0.060	0.99–1.38
Trig ≥ 1.7 mmol/L	1826	0.91	0.113	0.81–1.02	2625	1.12	0.106	0.98–1.28
Statin	2597	0.59	< 0.001	0.54–0.65	3159	0.54	< 0.001	0.49–0.61
School 12–15 years		0.82	< 0.001	0.75–0.89		0.83	0.001	0.74–0.93
School ≥ 15 years		0.72	0.011	0.55–0.92		0.65	0.043	0.42–0.99

Notes: N = total numbers in the specific age and sex group, C = cases (died in the study period more than one year after inclusion) and n = numbers in each LDL-C or numbers receiving statin after inclusion. ^1^Trig group < 1.0 mmol/L is the reference group and only the numbers in this group are therefore reported.

For triglycerides, the findings were almost the opposite. In females there was an increased mortality in the age group 50–60 years in all groups with a triglyceride level above 1 mmol/L. In the age group 60–70 years, there was an increased mortality in the group with the highest triglyceride level in both sexes.

There was a significant interaction between statin prescription and TC in both sexes in the age group 60–70 years, meaning that the frequency of statin use, as expected, was higher in the groups with higher TC. Furthermore, there was a significant interaction between HDL-C and statin use in females aged 60–70 years. No other interactions were found and the analyses regarding TC and HDL-C levels were adjusted for this interaction. Use of statin (after test date) and above 12 years of education provided a survival benefit that was significant in almost all age and lipoprotein/triglyceride groups in both sexes.

## Discussion

The current study aimed to test whether having levels of TC and its subfractions within the recommended target goals at baseline provided a survival difference in a general population of older adults. The most striking finding was that compared with the reference levels, high TC, HDL-C, or LDL-C levels were associated with lower mortality in the elderly and this was the case for even very high levels. The finding that high TC or LDL-C levels were associated with a lower mortality is contrary to the general assumption that there is a higher mortality among subjects with high lipoprotein levels. Our findings could seem controversial. However, most studies performed in older adults show an inverse association between TC and mortality [11,14] and a recent study demonstrated an inverse association between TC and non-cardiovascular mortality in a population free of CVD and statin use at baseline [13]. These findings were significant from the age of 65 years and were largely due to an inverse association with non-HDL-C. Our study demonstrates the same significant inverse association between high levels of TC and its subfractions and mortality in all age groups from as early as 50 years.

The evidence for a causal relationship between hyperlipidaemia and development of CVD is overwhelming [2,24]. However, our data indicate that a major part of the general population aged 50 + has lipoprotein levels above the recommended levels and that those groups without CVD or diabetes at measurement have a survival benefit compared with the groups with the recommended low levels.

The findings in triglycerides were contrary to the findings for TC, LDL-C, and HDL-C. There was a clear significant association between triglyceride levels and mortality risk in females but this was not as clear in males. Our study indicates that high triglyceride levels could be an important marker for decreased survival in females but not in males. A few previous studies have also indicated that elevated non-fasting triglycerides may be associated with increased risk of death in both sexes but most significantly in females [25–27]. However, a recent study did demonstrate a significant relation between non-fasting triglycerides and coronary heart disease mortality in men after more than 12 years of follow-up [3]. Our study has a shorter follow-up period, which could explain the lack of significance in males. Accordingly increased mortality among subjects with high triglyceride levels may be due to undiagnosed CVD or indicate that high triglyceride levels are associated with other harmful conditions.

Our data show that statin prescription after the first lipoprotein measurement provides a significant survival benefit in almost all age groups in both sexes irrespective of baseline lipid level. Placebo-controlled studies and meta-analyses have indicated that in patients with manifest or at risk of CVD, statin therapy is associated with a reduction in risk of heart attack, all-cause mortality, cardiovascular mortality, and ischaemic stroke [16,20,21]. However, there are only sparse data on statin therapy being beneficial for primary prevention, although several recent studies have reported mortality reductions with the use of statins for primary prevention [17,28–30]. A very recent Cochrane report concluded that evidence available to date showed that primary prevention with statins is likely to be cost-effective and may improve patients’ quality of life [31].Our data suggest that the beneficial effects of statin are partially independent of baseline lipid levels, as also reported in other studies [16,19].

There are some limitations in our study that should be taken into account when interpreting the results. The current study is based on requested lipoprotein analysis. These analyses should be requested for a specific reason and only in subjects where the requesting doctor is considering relevant action, for instance to initiate cholesterol-lowering treatment. As a consequence, subjects without known CVD risk factors or subjects with severe diseases, where cholesterol-lowering treatment is irrelevant, could be underrepresented in this population. However, we believe that our study population represents exactly the kind of general population to whom the target goals are addressed.

Second, the higher mortality in the reference group for TC and its subfractions could be due to a TC-lowering effect of severe disease. Multiple studies have demonstrated that low TC levels are associated with an increased risk of all-cause mortality [13,32]. We excluded all subjects with a survival shorter than one year after blood sampling to exclude all terminally ill subjects. However, it is possible that a fraction of the subjects with low TC levels may have severe diseases that are associated with a higher mortality. Although this group probably represents only a small fraction of all persons with low TC levels, substantially higher mortality rates among them might well increase the mortality rates of those with low TC considered as a group. The low TC levels at entry could reflect levels recently fallen from much higher levels with onset of disease. However, it seems that low levels of TC and its subfractions could be markers of all-cause mortality in later life.

Third, our study is conducted in a population aged 50 + years. The findings that high levels of LDL-C or TC are not associated with increased mortality could be due to selective survival, meaning that the subjects who are prone to the negative effects of high TC or LDL-C levels are already dead. The mortality is very low below the age of 50 years, which makes this explanation unlikely. However, we did exclude all those with a diagnosis of CVD or diabetes at baseline. These subjects could be the vulnerable subjects, leaving a population resistant to the harmful effects of high LDL-C or TC levels. A previous meta-analysis found that TC was positively correlated with ischaemic heart disease mortality even in the oldest [2]. This meta-analysis included studies with participants without previous disease at inclusion. We also excluded all those with a diagnosis of CVD or diabetes before or within 14 days after measurement. However, more than 11 000 individuals included in our study received a diagnosis of diabetes or CVD in the study period, who could be the vulnerable subjects. And still we see a survival benefit in the group with a TC and LDL-C above the recommended levels.

Finally, it is possible that our findings are influenced by residual confounding. We have tried to account for the known confounders age and sex by conducting the analysis in sex-specific age intervals. Furthermore, we have included statin use and educational status in our analysis. However, there may be other confounders that influenced our results. It is possible, for example, that individuals with a high TC measurement receive more medical attention and perhaps a different treatment, which influence mortality.

In conclusion, our population-based study shows that high TC, HDL-C, or LDL-C levels in the elderly are associated with a lower all-cause mortality compared with the group with the recommended low lipoprotein level. These findings are in opposition to the recommendation that in older adults without diabetes or CVD, lipoprotein values should be below specific values. In the current study higher lipoprotein levels do not seem to influence total mortality negatively. The opposite is the case for triglycerides, where the recommended low level is associated with lower all-cause mortality, especially in women. However, cholesterol-lowering treatment in the form of statins provides a survival benefit without correlation to cholesterol level. These findings could be considered in future recommendations for lipoprotein levels in elderly people without CVD or diabetes where the focus perhaps should change from lipoprotein levels to an increased focus on triglycerides.
